# Flow cytometry defined cytoplasmic immunoglobulin index is a major prognostic factor for progression of asymptomatic monoclonal gammopathies to multiple myeloma (subset analysis of SWOG S0120)

**DOI:** 10.1038/bcj.2016.19

**Published:** 2016-03-25

**Authors:** X Papanikolaou, A Rosenthal, M Dhodapkar, J Epstein, R Khan, F van Rhee, Y Jethava, S Waheed, M Zangari, A Hoering, J Crowley, D Alapat, F Davies, G Morgan, B Barlogie

**Affiliations:** 1 Myeloma Institute for Research and Therapy, University of Arkansas for Medical Sciences, Little Rock, AR, USA; 2 Cancer Research and Biostatistics, Seattle, WA, USA; 3 Yale School of Medicine, New Haven, CT, USA; 4 Department of Pathology, University of Arkansas for Medical Sciences, Little Rock, AR, USA

Multiple myeloma (MM) is a clonal plasma cell (PC) disorder characterized by end organ damage that is in turn characterized by CRAB criteria (calcium and creatinine elevation, anemia and bone lesions).^1^ It is commonly accepted that nearly all cases of MM are preceded by a clinically benign phase of monoclonal gammopathy of undetermined significance (MGUS) that evolves through a stage of smoldering multiple myeloma (SMM) without end organ damage,^2^ collectively referred to as asymptomatic monoclonal gammopathies (AMG).^3^ Although traditionally SMM is considered more prone to MM progression than MGUS, additional variables, such as involved-to-uninvolved free light-chain ratio^4^ and magnetic resonance imaging-defined focal lesion number and size,^5^ have been linked to progression to MM and form the basis for the newest International Myeloma Working Group criteria for MM.^6^ As the treatment of MM has been greatly advanced, emphasis has been placed on identifying patients with AMG at high risk of progression to MM so that, with earlier treatment, end organ damage can be minimized.^7^ Many new high-risk variables have indeed been identified such as level of circulating plasma cells^8^ and gene expression profiling (GEP).^9, 10^

We have previously reported that two-parameter flow cytometry of DNA and cytoplasmic light-chain immunoglobulin (DNA/CIG) is highly predictive of progression-free and overall survival in newly diagnosed MM treated with Total Therapy.^11^ In the current subset analysis of S0120, we have investigated whether the DNA/CIG assay can also identify patients with AMG at high risk for progression to MM requiring therapy (time to therapy, TTT).^12^ Of 254 patients enrolled at the University of Arkansas in the observational SWOG S0120 protocol with AMG, 110 had evaluable DNA/CIG information and retained AMG status according to the revised International Myeloma Working Group criteria for MM.^6^ All patients underwent detailed clinical staging as previously reported.^9, 10^ DNA/CIG assay was performed on whole bone marrow aspirates along with metaphase cytogenetics and GEP of CD138+ purified PC.^13^ Imaging studies involved metastatic bone surveys and, in the majority of the cases, magnetic resonance imaging examination of the axial and appendicular skeleton.

Details of the DNA/CIG method have been published elsewhere.^14, 15^ A technical modification of the assay was applied uniformly since August 2006. The assay is based on the two-parameter flow cytometry of cytoplasmic immunoglobulin and DNA of whole bone marrow aspirates. Single-cell suspensions were exposed to anti-light-chain reagents (Dako Kappa and Lambda light chain F(AB)_2_/FITC conjugated) and then counterstained for DNA with propidium iodide with the addition of RNase. To quantitate the cellular DNA content, DNA index (DI)^16^ was determined and calculated as the ratio of the mean for each light-chain-positive G0/1 DNA peak divided by the mean of the light-chain-negative diploid G0/1 peak on the *X axis*. A DI between 0.99 and 1.01 was referred to as diploid, while hyperdiploid implied DI>1.01 and hypodiploid DI<0.99. The excess of kappa- or lambda-positive cells identified the involved or light-chain-restricted (LCR) cell population, the percentage of which was calculated in relation to the total number of gated events. Among the LCR cell population, discrete populations of cells with different DI were identified, which we refer to from here on as DNA stem lines. The involved DNA stem line with the highest percentage was considered dominant. To quantitate the cytoplasmic immunoglobulin content of a light-chain-positive population, the cytoplasmic immunoglobulin index (CIg) was used and calculated from the ratio of the geometric mean of the *Y axis* (cytoplasmic immunoglobulin fluorescence intensity) for the light-chain-positive G0/1 peak divided by the *Y axis* geometric mean of the light-chain-negative diploid G0/1 population. The CIg of each distinct DNA stem line was calculated as explained above.

Kaplan–Meier methods were used to generate survival distribution graphs, and comparisons were made employing the log-rank test. For continuous variables, the running log-rank method was applied for the calculation of optimal cutoff points. The *R*^2^ statistic was used to evaluate the predictive power of different models. Wilcoxon tests were used to compare the medians of continuous measurements between groups.

The characteristics of the 110 patients lacking the revised International Myeloma Working Group criteria for MM are portrayed in Supplementary Table 1. The median follow-up time for the 110 patients was 4.8 years. Aneuploidy by DNA/CIG was evident in 64%, all of whom had hyperdiploid stem lines, while additional hypodiploid abnormalities were present in two cases. Low hemoglobin (<10 g/dl) pertained to only 4% (non-plasma cell dyscrasia-related reasons) while creatinine ⩾2 mg/dl was evident in one case due to hypertension-related nephrosclerosis. Metaphase cytogenetic abnormalities (CA) were documented in 16%, a GEP70 score⩾−0.26^(ref. 3)^ pertained to 33% and a recently defined novel GEP4 score⩾9.28^(ref. 17^) to 12% of patients.

We examined the TTT probability of AMG (Table 1). Optimal cutoff points were obtained for all continuous numerical values. We confirm other studies linking older age ⩾65 years, albumin <3.5 g/dl, B2M⩾3.5 mg/l, serum-M⩾3 g/dl and bone marrow plasmacytosis ⩾10%^(refs 3,17)^ to TTT for MM, along with an involved-to-uninvolved free light-chain ratio >8.^4^ The presence of CA, GEP70- and GEP4- high-risk designations was strongly linked to inferior TTT. Among DNA/CIG-derived parameters, CIg<3.6 and LCR% >17 were both strongly linked to progression to MM. Other DNA/CIG variables associated with TTT included the presence of aneuploidy and the presence of ⩾2 DNA stem lines (Figure 1). The 26 patients with CIg<3.6 had a 2-year TTT probability of 55.2% compared with 7.1% among the remaining 84 with higher values (Figure 1a). Similarly, higher LCR% >17 present in 20 patients conferred a 2-year MM progression rate of 60% versus 9% among the 90 with lower (Figure 1b). Consideration of both DNA/CIG features identified 14 patients displaying two high-risk features with 2-year TTT of 71.4% as opposed to 5.1% in 78 patients with only favorable features, while the presence of one adverse variable present in 18 patients was associated with a 2-year TTT probability of approximately 34% (Figure 1c).

In the multivariate model, serum-M⩾3 g/dl, CIg<3.6 and LCR>17% independently conferred adverse outcomes (Table 1). All three parameters combined provided for a high *R*^2^ value of 0.861, implying that TTT probability could be accounted for in 86% (Supplementary Table 2). In comparison, the classical criteria of bone marrow plasmacytosis ⩾10% and serum-M⩾3 g/dl had a lower cumulative *R*^2^ of 0.632.

When only the sub-population of SMM (80 patients; Supplementary Table 3) was considered, DNA/CIG-derived variables retained their statistical significance (Supplementary Table 4). Both LCR>17% and CIg<3.6 identified 14 patients with a 71% 2-year TTT probability as opposed to 6% for the 50 patients with only favorable features; the presence of one adverse feature, present in 16 patients, was associated with a TTT probability of approximately 38% (Figure 1d). The multivariate model for this cohort of patients (without GEP variables) included CIg<3.6, LCR>17% and serum-M⩾3 g/dl; albumin<3.5 g/dl and B2M⩾3.5 mg/l also conferred higher TTT probability for a *R*^2^ of 0.862 (Supplementary Tables 4 and 5). The inclusion of GEP variables, available in a subset of 61 patients, identified GEP-4 as a significant variable, dispelling CIg and B2M from the model (*R*^2^=0.895; Supplementary Tables 4 and 6).

CIg is a measure of plasma cell immunoglobulin production.^15^ We therefore examined CIg values in patients with MGUS and SMM (both from the S0120 trial), and in newly diagnosed MM patients accrued to Total Therapy 3b.^18^ Median CIg values declined progressively with the transition from MGUS to SMM and later to MM (10.5 versus 5.6 versus 3.3, *P*<0.001; Supplementary Figure 1a). To exclude the possibility that the difference in CIg reflects the decreasing percentage of highly secreting normal plasma cells with the evolution of plasma cell dyscrasias,^19, 20^ the analysis was repeated for strictly aneuploid cases. Again, the evolution from MGUS to SMM to MM was characterized by a progressively lower CIg (16.0 versus 9.1 versus 3.5, *P*<0.0001; Supplementary Figure 1b).

In summary, DNA/CIG offers powerful prognostic information for AMG even in the era of genomic profiling. While LCR% reflects tumor burden, the finding of progressively decreasing CIg with the evolution of plasma cell dyscrasias in this single institution subset analysis of S0120 is novel. It provides evidence that the progression of plasma cell dyscrasias is accompanied by a progressive decline in immunoglobulin production capacity.

## Figures and Tables

**Figure 1:**
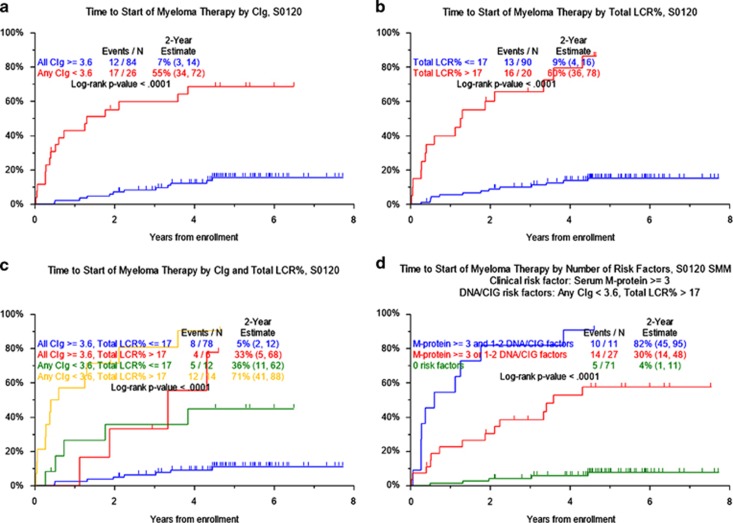
Kaplan–Meier plots for the time to progression from AMG to MM requiring therapy according to: CIg, (**a**) total LCR%, (**b**) the combination of CIg and total LCR% (**c**) and the combination of CIg and total LCR% for the SMM population (**d**).

**Table 1:** Cox regression for time to progression to MM

*Variables*	n*/*N *(%)*	*Time to treatment for MM*	
		*HR (95% CI)*	P*-value*
*Univariate*			
Age ⩾65 years	49/110 (45%)	3.49 (1.58, 7.69)	<0.001
Female	52/110 (47%)	0.67 (0.32, 1.42)	0.295
White	91/110 (83%)	7.11 (0.97, 52.24)	0.024
Albumin<3.5 g/dl	21/110 (19%)	4.39 (2.10, 9.15)	<0.001
B2M ⩾3.5 mg/l	32/109 (29%)	2.85 (1.37, 5.92)	0.003
B2M>5.5 mg/l	6/109 (6%)	1.54 (0.37, 6.50)	0.552
Creatinine ⩾2 mg/dl	1/110 (1%)	0.00 (0.00)	0.562
CRP ⩾8 mg/l	28/110 (25%)	0.83 (0.34, 2.04)	0.685
Hb<10 g/dl	4/110 (4%)	4.23 (1.26, 14.14)	0.011
LDH ⩾190 U/l	12/110 (11%)	0.69 (0.16, 2.91)	0.614
M Protein ⩾3 g/dl	17/109 (16%)	7.52 (3.58, 15.82)	<0.001
BMPC ⩾10%	72/110 (65%)	5.43 (1.64, 17.96)	0.002
Involved light chain>10 mg/dl	32/86 (37%)	1.56 (0.73, 3.34)	0.248
Involved/uninvolved ratio>8	45/86 (52%)	2.51 (1.10, 5.75)	0.024
Cytogenetic abnormalities	18/110 (16%)	3.83 (1.77, 8.27)	<0.001
GEP 70-gene risk>−0.26	28/84 (33%)	6.39 (2.59, 15.77)	<0.001
GEP 4-gene score⩾9.28	10/84 (12%)	7.57 (3.14, 18.26)	<0.001
Number of stem lines⩾2	64/110 (58%)	3.29 (1.34, 8.10)	0.006
Any aneuploidy	70/110 (64%)	2.52 (1.03, 6.19)	0.037
Total LCR% >17	20/110 (18%)	10.92 (5.14, 23.16)	<0.001
Any CIg<3.6	26/110 (24%)	7.90 (3.75, 16.66)	<0.001

*Multivariate*			
M Protein ⩾3 g/dl	17/109 (16%)	4.57 (2.08, 10.04)	<0.001
Total LCR% >17	20/109 (18%)	4.72 (2.07, 10.76)	<0.001
Any CIg<3.6	26/109 (24%)	3.97 (1.75, 8.99)	<0.001

Abbreviations: BMPC, bone marrow plasma cells; CI, confidence interval; CIg, cytoplasmic immunoglobulin index; CRP, C-reactive protein; GEP, gene expression profiling; HR, hazard ratio; LCR, light chain restricted; LDH, lactate dehydrogenase; MM, Multiple myeloma, NS2, not significant at 0.005 level. *P*-value from score *χ*^2^-test in Cox regression. NS2- Multivariate results not statistically significant at 0.05 level. All univariate *P*-values reported regardless of significance. Multivariate model uses stepwise selection with entry level 0.1 and variable remains if it meets the 0.05 level. A multivariate *P*-value >0.05 indicates variable forced into model with significant variables chosen using stepwise selection.
