# Supplementary material for: Flow cytometry defined cytoplasmic immunoglobulin index is a major prognostic factor for progression of asymptomatic monoclonal gammopathies to multiple myeloma (subset analysis of SWOG S0120)
Source: Blood Cancer J. 2016 Mar 25;6(3):e410–. doi: 10.1038/bcj.2016.19 (PMC4817101; doi:10.1038/bcj.2016.19)

**Supplemental Table 1: Characteristics of patients with AMG**

| **Factor** | **n/N (%)** |
| --- | --- |
| Median Age (Yrs) | 63.8 (N=110) (37.9 - 91.2) |
| Age >= 65 yr | 49/110 (45%) |
| Female | 52/110 (47%) |
| White | 91/110 (83%) |
| Albumin < 3.5 g/dL | 21/110 (19%) |
| B2M >= 3.5 mg/L | 32/109 (29%) |
| B2M > 5.5 mg/L | 6/109 (6%) |
| Creatinine >= 2 mg/dL | 1/110 (1%) |
| CRP >= 8 mg/L | 28/110 (25%) |
| Hb < 10 g/dL | 4/110 (4%) |
| LDH >= 190 U/L | 12/110 (11%) |
| M Protein >= 3 g/dL | 17/109 (16%) |
| BMPC >= 10% | 72/110 (65%) |
| Involved light chain > 10 (mg/dL) | 32/86 (37%) |
| Involved/uninvolved ratio > 8 | 45/86 (52%) |
| Cytogenetic abnormalities | 18/110 (16%) |
| GEP 70-gene risk > -0.26 | 28/84 (33%) |
| GEP 4-gene score >= 9.28 | 10/84 (12%) |
| Number of stem lines >= 2 | 64/110 (58%) |
| Any hyperdiploid | 70/110 (64%) |
| Any hypodiploid | 2/110 (2%) |
| Any aneuploidy | 70/110 (64%) |
| Total LCR% > 17 | 20/110 (18%) |
| Any CIg < 3.6 | 26/110 (24%) |
| n/N (%):  n- Number with factor, N- Number with valid data for factor ND: No valid observations for factor | |

**Supplemental Table 2: Cumulative R^2^ for multivariate model for AMG**

|  |  | **Time to Therapy for MM** | |  |
| --- | --- | --- | --- | --- |
| **Variable** | **n/N (%)** | **HR (95% CI)** | **P-value** | **Cumulative R^2^** |
| Total LCR% > 17 | 20/109 (18%) | 4.72 (2.07, 10.76) | <.001 | 0.697 |
| M Protein >= 3 g/dL | 17/109 (16%) | 4.57 (2.08, 10.04) | <.001 | 0.798 |
| Any CIg < 3.6 | 26/109 (24%) | 3.97 (1.75, 8.99) | <.001 | 0.861 |

**Supplemental Table 3: Characteristics of patients with SMM**

| **Factor** | **n/N (%)** |
| --- | --- |
| Median Age (Yrs) |  |
| Age >= 65 yr | 36/80 (45%) |
| Female | 35/80 (44%) |
| White | 68/80 (85%) |
| Albumin < 3.5 g/dL | 19/80 (24%) |
| B2M >= 3.5 mg/L | 28/79 (35%) |
| B2M > 5.5 mg/L | 6/79 (8%) |
| Creatinine >= 2 mg/dL | 1/80 (1%) |
| CRP >= 8 mg/L | 19/80 (24%) |
| Hb < 10 g/dL | 4/80 (5%) |
| LDH >= 190 U/L | 9/80 (11%) |
| M Protein >= 3 g/dL | 17/79 (22%) |
| BMPC >= 10% | 66/80 (83%) |
| Involved light chain > 10 (mg/dL) | 27/68 (40%) |
| Involved/uninvolved ratio > 8 | 37/68 (54%) |
| Cytogenetic abnormalities | 17/80 (21%) |
| GEP 70-gene risk > -0.26 | 22/61 (36%) |
| GEP 4-gene score >= 9.28 | 8/61 (13%) |
| Number of stem lines >= 2 | 49/80 (61%) |
| Any hyperdiploid | 53/80 (66%) |
| Any hypodiploid | 2/80 (3%) |
| Any aneuploidy | 53/80 (66%) |
| Total LCR% > 17 | 19/80 (24%) |
| Any CIg < 3.6 | 25/80 (31%) |
| n/N (%):  n- Number with factor, N- Number with valid data for factor ND: No valid observations for factor | |

**Supplemental Table 4: Cox regression for time to progression to MM, SMM patients**

|  | | | **Time to treatment for MM** | |  |
| --- | --- | --- | --- | --- | --- |
|  | **Variable** | **n/N (%)** | **HR (95% CI)** | **P-value** |  |
| Univariate | Age >= 65 yr | 36/80 (45%) | 3.88 (1.69, 8.92) | <.001 |  |
|  | Female | 35/80 (44%) | 0.87 (0.40, 1.88) | 0.728 |  |
|  | White | 68/80 (85%) | 5.79 (0.78, 42.68) | 0.051 |  |
|  | Albumin < 3.5 g/dL | 19/80 (24%) | 3.80 (1.78, 8.11) | <.001 |  |
|  | B2M >= 3.5 mg/L | 28/79 (35%) | 2.60 (1.21, 5.56) | 0.011 |  |
|  | B2M > 5.5 mg/L | 6/79 (8%) | 1.12 (0.27, 4.74) | 0.876 |  |
|  | Creatinine >= 2 mg/dL | 1/80 (1%) | 0.00 (0.00, .) | 0.501 |  |
|  | CRP >= 8 mg/L | 19/80 (24%) | 1.13 (0.45, 2.80) | 0.798 |  |
|  | Hb < 10 g/dL | 4/80 (5%) | 3.00 (0.89, 10.05) | 0.062 |  |
|  | LDH >= 190 U/L | 9/80 (11%) | 0.74 (0.18, 3.12) | 0.680 |  |
|  | M Protein >= 3 g/dL | 17/79 (22%) | 5.45 (2.54, 11.70) | <.001 |  |
|  | BMPC >= 10% | 66/80 (83%) | 3.12 (0.74, 13.16) | 0.103 |  |
|  | Involved light chain > 10 (mg/dL) | 27/68 (40%) | 1.45 (0.67, 3.14) | 0.342 |  |
|  | Involved/uninvolved ratio > 8 | 37/68 (54%) | 2.15 (0.93, 4.95) | 0.066 |  |
|  | Cytogenetic abnormalities | 17/80 (21%) | 3.09 (1.41, 6.79) | 0.003 |  |
|  | GEP 70-gene risk > -0.26 | 22/61 (36%) | 7.69 (2.89, 20.45) | <.001 |  |
|  | GEP 4-gene score >= 9.28 | 8/61 (13%) | 10.84 (4.21, 27.89) | <.001 |  |
|  | Number of stem lines >= 2 | 49/80 (61%) | 3.50 (1.32, 9.24) | 0.007 |  |
|  | Any aneuploidy | 53/80 (66%) | 2.71 (1.03, 7.17) | 0.036 |  |
|  | Total LCR% > 17 | 19/80 (24%) | 8.06 (3.68, 17.65) | <.001 |  |
|  | Any CIg < 3.6 | 25/80 (31%) | 5.98 (2.72, 13.13) | <.001 |  |
| Multivariate, No GEP | Albumin < 3.5 g/dL | 19/78 (24%) | 2.50 (1.01, 6.16) | <.001 |  |
|  | B2M >= 3.5 mg/L | 28/78 (36%) | 2.74 (1.08, 7.00) | 0.033 |  |
|  | M Protein >= 3 g/dL | 17/78 (22%) | 4.22 (1.84, 9.69) | <.001 |  |
|  | Total LCR% > 17 | 19/78 (24%) | 5.78 (2.46, 13.60) | <.001 |  |
|  | Any CIg < 3.6 | 25/78 (32%) | 3.39 (1.45, 7.95) | 0.008 |  |
| Multivariate, GEP | Albumin < 3.5 g/dL | 13/60 (22%) | 5.03 (1.89, 13.34) | <.001 |  |
|  | M Protein >= 3 g/dL | 14/60 (23%) | 3.74 (1.40, 9.98) | 0.006 |  |
|  | GEP 4-gene score >= 9.28 | 8/60 (13%) | 5.17 (1.78, 14.99) | <.001 |  |
|  | Total LCR% > 17 | 16/60 (27%) | 5.90 (2.21, 15.71) | .001 |  |
| HR- Hazard Ratio, 95% CI- 95% Confidence Interval, P-value from Score Chi-Square Test in Cox Regression NS2- Multivariate results not statistically significant at 0.05 level. All univariate p-values reported regardless of significance. Multivariate model uses stepwise selection with entry level 0.1 and variable remains if meets the 0.05 level. A multivariate p-value greater than 0.05 indicates variable forced into model with significant variables chosen using stepwise selection. | | | | |  |

**Supplemental Table 5: Cumulative R^2^ for multivariate model w/o GEP for SMM patients**

|  |  | **Time to Therapy for MM** | |  |
| --- | --- | --- | --- | --- |
| **Variable** | **n/N (%)** | **HR (95% CI)** | **P-value** | **Cumulative R^2^** |
| Total LCR% > 17 | 19/78 (24%) | 5.78 (2.46, 13.60) | <.001 | 0.599 |
| Albumin < 3.5 g/dL | 19/78 (24%) | 2.50 (1.01, 6.16) | <.001 | 0.716 |
| M Protein >= 3 g/dL | 17/78 (22%) | 4.22 (1.84, 9.69) | <.001 | 0.818 |
| Any CIg < 3.6 | 25/78 (32%) | 3.39 (1.45, 7.95) | 0.008 | 0.859 |
| B2M >= 3.5 mg/L | 28/78 (36%) | 2.74 (1.08, 7.00) | 0.033 | 0.862 |

**Supplemental Table 6: Cumulative R^2^ for multivariate model w/ GEP for SMM patients**

|  |  | **Time to Therapy for MM** | |  |
| --- | --- | --- | --- | --- |
| **Variable** | **n/N (%)** | **HR (95% CI)** | **P-value** | **Cumulative R^2^** |
| GEP 4-gene score >= 9.28 | 8/60 (13%) | 5.17 (1.78, 14.99) | <.001 | 0.610 |
| Albumin < 3.5 g/dL | 13/60 (22%) | 5.03 (1.89, 13.34) | <.001 | 0.761 |
| Total LCR% > 17 | 16/60 (27%) | 5.90 (2.21, 15.71) | .001 | 0.854 |
| M Protein >= 3 g/dL | 14/60 (23%) | 3.74 (1.40, 9.98) | 0.006 | 0.895 |

**Supplemental Figure 1** Boxplots of dominant CIg values for MGUS and SMM of S0120; and TT3b MM, in all cases (a), and in only aneuploid dominant stem lines (b)

**Supplemental Figure 1a**


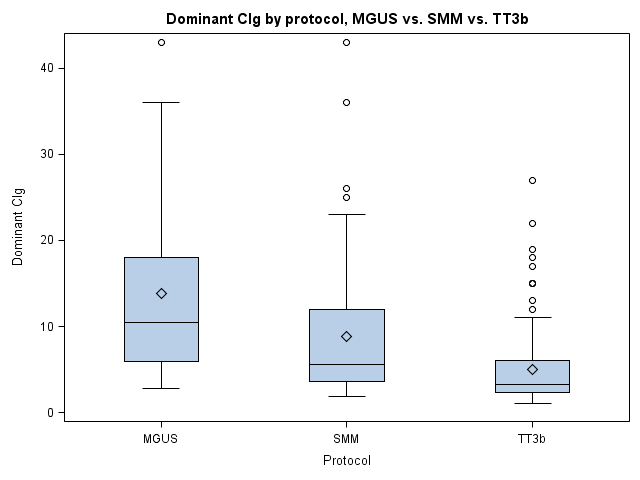


**Supplemental Figure 1b**


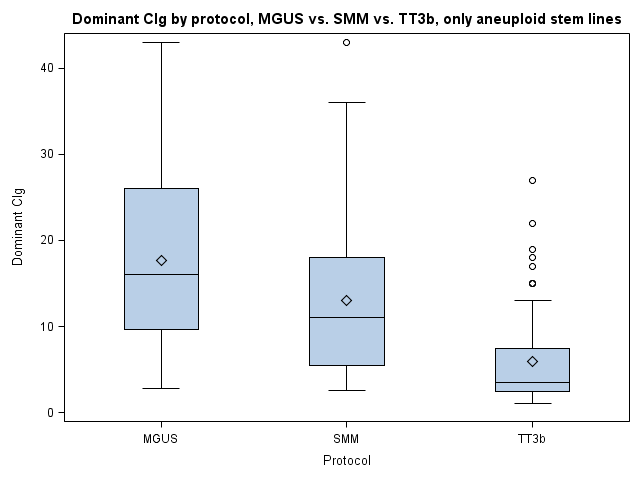

Supplement: Supplementary Information [file bcj201619x1.docx]
